# Evaluation of Implementation of Newborn Screening for Sickle Cell Disease Program in Selected Hospitals in Dar es Salaam, Tanzania

**DOI:** 10.3390/ijns12020024

**Published:** 2026-04-15

**Authors:** Tunganege Matipa, Elia Nyangi, Agnes Jonathan, Mwashungi Ally, Lulu Chirande, Asteria Mpoto, Emmanuel Balandya, Gladys Reuben Mahiti

**Affiliations:** 1 Department of Development Studies, School of Public Health and Social Sciences, Muhimbili University of Health and Allied Sciences, Dar es Salaam P.O. Box 65001, Tanzania; nyangielia@gmail.com (E.N.); agnesjonathan4@gmail.com (A.J.); gmahiti2011@gmail.com (G.R.M.); 2 Sickle Pan African Research Consortium (SPARCO) Tanzania, Department of Haematology and Blood Transfusion, School of Diagnostic Medicine, Muhimbili University of Health and Allied Sciences, Dar es Salaam P.O. Box 65001, Tanzania; mwashungi.ally@muhas.ac.tz (M.A.); chirandelulu@yahoo.com (L.C.); ebalandya@yahoo.com (E.B.); 3 Department of Haematology and Blood Transfusion, School of Diagnostic Medicine, College of Medicine, Muhimbili University of Health and Allied Sciences, Dar es Salaam P.O. Box 65001, Tanzania; 4 Department of Paediatrics and Child Health, School of Clinical Medicine, College of Medicine, Muhimbili University of Health and Allied Sciences, Dar es Salaam P.O. Box 65001, Tanzania; 5 Sickle Cell Disease Unit, Non-Communicable Diseases Section, Directorate of Curative Service, Ministry of Health, Dodoma P.O. Box 743, Tanzania; mpotoaster@gmail.com; 6 Department of Physiology, School of Biomedical Sciences, College of Medicine, Muhimbili University of Health and Allied Sciences, Dar es Salaam P.O. Box 65001, Tanzania

**Keywords:** sickle cell disease, newborn screening, comprehensive care, implementation, Tanzania, process evaluation

## Abstract

Sickle cell disease (SCD) is a major public health concern in Tanzania where approximately 11,000 children are born with the condition annually. Newborn screening (NBS) enables early diagnosis and timely intervention. Despite the proven effectiveness of NBS in reducing early mortality from SCD, implementation in Tanzania remains limited to pilot programs at facilities such as Temeke and Amana Regional Referral Hospitals (RRHs) in the city of Dar-es-salaam. This study evaluated the implementation of NBS for the SCD Program at Temeke and Amana RRHs. An explanatory mixed-methods process evaluation was conducted between January 2022 and December 2024. Quantitative data were extracted from hospital registries and REDCap, while qualitative data were obtained from key informant interviews with 17 healthcare workers. Quantitative data were analyzed using SPSS v29.0, while qualitative transcripts were thematically analyzed using NVivo software version 15 to explore operational factors influencing implementation. A total of 10,711 newborns were screened across the two hospitals. Seventy-four (0.70%) newborns had homozygous SCD (HbS/S), whereas 1325 (12.53%) had sickle cell trait (HbA/S). Enrolment of infants diagnosed with SCD into comprehensive care declined substantially over time, from 65.6% in 2022 to 10.5% in 2024 at Temeke RRH, while Amana RRH recorded no enrolments beyond the first year of implementation. Qualitative findings highlighted facilitators for NBS such as maternal awareness, interdepartmental collaboration, and the availability of trained staff. However, implementation was hindered by inadequate refresher training, delayed staff incentives, supply shortages, and parental hesitancy influenced by cultural beliefs. This evaluation found a substantial decline in enrolment of newborns diagnosed with SCD into comprehensive care, driven by key operational challenges. Although early implementation benefited from trained, committed staff and interdepartmental collaboration, sustainability was limited by inadequate refresher training, delayed incentives, supply shortages, and parental hesitancy. Addressing these gaps through regular capacity building, strengthened supply chains, timely incentives, and culturally sensitive community education is critical to improving enrolment, continuity of care, and informing national scale-up of NBS for SCD in Tanzania.

## 1. Introduction

Sickle cell disease (SCD), a disorder resulting from the inheritance of abnormal sickle haemoglobin (HbS) in either homozygous form (HbS/S), compound heterozygosity with haemoglobin C or D (HbS/C, HbS/D) or co-inheritance of HbS with beta thalassemia (HbS/beta thal), is a life-threatening inherited blood disorder characterized by severe anemia, recurrent pain, and progressive end-organ damage, leading to reduced life expectancy. Globally, more than 500,000 children are born with SCD each year, with Sub-Saharan Africa (SSA) accounting for over 75% of cases, a figure projected to reach 85% by 2050 [1]. Tanzania is among the top five most affected countries in the world, with an estimated 11,000–15,000 newborns diagnosed annually [2]. SCD contributes to 7% of under-five mortality in Tanzania, making it a major public health concern. In recognition of this burden, the World Health Organization (WHO) designated SCD a priority condition in 2006, and Tanzania subsequently incorporated SCD management into its National Non-Communicable Disease Strategy [3]. Despite these efforts, challenges such as late diagnosis, limited access to comprehensive care, and inadequate community awareness continue to fuel high mortality rates across SSA.

Newborn screening (NBS) for SCD has emerged as a crucial public health intervention, enabling early diagnosis and timely initiation of life-saving measures such as prophylactic antibiotics, pneumococcal vaccination, folic acid, hydroxyurea, parental education, and regular clinical monitoring. Evidence from both high-income and resource-limited settings shows that NBS and early enrolment in comprehensive care significantly reduce morbidity and mortality among affected children [4,5]. In countries such as the United States and the United Kingdom, survival into adulthood for individuals with SCD exceeds 90% following the introduction of NBS and comprehensive care programs [6]. Similarly, pilot NBS programs in Africa, including Ghana, have demonstrated feasibility and effectiveness in improving early access to care [5,7]. In Tanzania, NBS has been piloted in selected regions such as Dar es Salaam and Mwanza under the Ministry of Health’s Non-Communicable Diseases framework, with technical support from the Sickle Cell Program at Muhimbili University of Health and Allied Sciences (MUHAS) and partnerships with international collaborators [8,9]. The program integrates screening into existing maternal and child health services, using heel-prick blood sampling for screening using Isoelectric Focusing followed by confirmatory testing using High Performance Liquid Chromatography for infants with homozygous SCD (HbS/S) and compound heterozygous states, or molecular diagnosis using Oxford Nanopore Technology for infants who are suspected to have HbS/beta thal. Confirmed cases are referred for enrolment in comprehensive care programs, which provide preventive and supportive care. While these initiatives have demonstrated promise, systemic challenges persist. These include inconsistent adherence to screening protocols, inadequate training of healthcare workers, logistical difficulties in sample collection and transport, and weak linkage of diagnosed infants to long-term care [8,9].

Despite Tanzania’s high burden of SCD and the demonstrated potential of NBS, its integration into routine healthcare services remains at an early stage. Dar es Salaam, the country’s largest city with some of the busiest health facilities, provides an ideal context to evaluate implementation. However, there is limited evidence on the coverage, effectiveness, and operational factors of NBS for SCD in this setting. This study aimed to evaluate the implementation of the NBS program for SCD in selected hospitals in Dar es Salaam, Tanzania, focusing on identifying its operational factors to generate evidence that can inform national policy and support program scale-up.

## 2. Methods

### 2.1. Study Setting

The study was conducted at Temeke Regional Referral Hospital (TRRH) and Amana Regional Referral Hospital (ARRH), two public regional referral hospitals in Dar es Salaam, Tanzania. These hospitals, located in Temeke and Ilala municipalities, assist an average of 1200–2400 deliveries per month, provide comprehensive maternal, newborn, and child health services, and possess the infrastructure and staffing necessary to support NBS activities [10,11]. Between January 2022 and December 2024, both hospitals participated in pilot NBS programs integrated into routine postnatal care. Blood samples were collected shortly after birth using dried blood spot (DBS), with initial screening conducted via Isoelectric Focusing (IEF) using Multiphor II Electrophoresis Unit (GE Healthcare Biosciences AB, Uppsala, Sweden) as per manufacturer’s standard operating procedures. This was followed by confirmatory hemoglobin testing via High Performance Liquid Chromatography using Biorad VARIANT nbs HPLC (Bio-Rad Laboratories, Inc., Hercules, CA, USA) for infants with homozygous SCD (HbS/S) and compound heterozygous states, or molecular diagnosis using ONT Nanopore MinION (Oxford Nanopore Technologies, Oxford, UK) for infants who are suspected to have HbS/beta thal. The NBS and follow-up involved nurse-midwives, laboratory personnel and clinicians, while pediatricians, medical officers, and nurses provided ongoing care at hospital-based SCD clinics. Both hospitals operate dedicated SCD clinics within paediatric outpatient departments, offering comprehensive care including routine physical checks, caregiver education, hydroxyurea, prophylactic medications, vaccination referral, malaria prevention, and management of SCD-related complications.

### 2.2. Study Design

An explanatory mixed-methods design was employed. Quantitative data assessed the proportions of live births, screened neonates, confirmed SCD cases, and enrolment into comprehensive care using hospital delivery registers, laboratory records, and clinic databases. Qualitative data explored healthcare providers’ experiences and contextual factors influencing NBS implementation. Guided by process evaluation principles, the study examined implementation reach and contextual influences, enabling integrated interpretation of outcome measures and implementation processes. Process evaluation helps to understand how and why interventions work within their implementation context and is particularly valuable for complex programs such as NBS, by assessing fidelity, reach, dose delivered, and contextual influences [12]. The mixed-methods approach enabled a comprehensive understanding of both measurable outcomes and implementation processes. Quantitative data, drawn from hospital records, evaluated the proportions of neonates born, screened, and enrolled into care between January 2022 and December 2024, while qualitative data explored healthcare providers’ experiences and contextual factors influencing NBS implementation.

### 2.3. Recruitment of Study Participants

Quantitative data were drawn from hospital birth records and the NBS REDCap database for all neonates born, screened for SCD and enrolled into comprehensive care during the study period. For the qualitative component, 17 healthcare workers directly involved in NBS implementation at the two hospitals were purposively selected and interviewed to explore their experiences and perspectives on program delivery.

### 2.4. Data Collection and Analysis

Data were collected using both quantitative and qualitative methods. Quantitative data were extracted from hospital birth records and the REDCap (Vanderbilt University Medical Center, Nashville, TN, USA, 2004) using standardized forms, aligned with Ministry of Health and World Health Organization (WHO) indicators to ensure validity and reliability, to determine the number of neonates born, screened, diagnosed, and enrolled in care between January 2022 and December 2024. Qualitative data were obtained through Key Informant Interviews (KII) with purposively selected healthcare workers directly involved in NBS activities, using semi-structured guides developed in English and translated into Kiswahili to explore facilitators, challenges, and recommendations for program improvement. Interviews were conducted by the corresponding author, in Kiswahili language. All interviews were audio-recorded. Each interview lasted 30–50 min and was conducted privately to ensure confidentiality. Quantitative data were analysed using SPSS v29.0 (IBM, New York, NY, USA, 2022) to determine the proportions of newborns screened and enrolled into care, while qualitative data were analysed using Graneheim and Lundman’s content analysis framework [13] with NVivo software version 15 (Lumivero, Denver, CO, USA, 2024) to identify codes, categories, and themes. The audio-recorded interviews were transcribed verbatim, and initial codes were discussed with the last author. Trustworthiness of qualitative findings was ensured through credibility, dependability, confirmability, and transferability measures, including triangulation with quantitative results. The integration of both datasets enabled a robust interpretation of program performance and contextual factors influencing NBS implementation.

### 2.5. Ethical Considerations

We obtained ethical approval from the Muhimbili University of Health and Allied Sciences (MUHAS) Research Ethics Committee (Ref: MUHAS-REC-03-2025-2696) and the hospital authorities at TRRH and ARRH. Written informed consent was obtained from all participants. Confidentiality and voluntary participation were ensured throughout the study.

## 3. Results

### 3.1. Quantitative Findings

#### 3.1.1. Trends in Proportions of Neonates Screened for SCD

**Figure 1:**
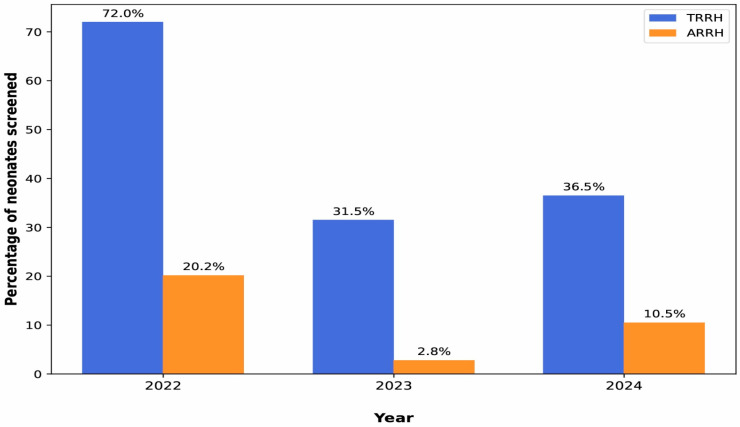
Proportion of livebirths screened for SCD at Amana and Temeke Regional Referral Hospitals, 2022–2024.

Both TRRH and ARRH experienced declining live births from 2022 to 2024, alongside substantial fluctuations and overall reductions in NBS for SCD. At TRRH, screening coverage dropped sharply from 72.5% in 2022 (4533 out of 6294 livebirths) to around one-third in 2024 (1867 out of 5110 livebirths), while at ARRH, it fell from 20.2% in 2022 (1539 out of 7617 livebirths) to 2.8% in 2023 (217 out of 7750 livebirths), with only partial recovery to 10.5% in 2024 (624 out of 5923 livebirths; Figure 1). The proportion of identified SCD cases remained low at both facilities but fluctuated over time. At TRRH, 32, 9 and 19 newborns were diagnosed with SCD while at ARRH, 7, 2 and 4 newborns were diagnosed with SCD in 2022, 2023 and 2024, respectively. All cases were homozygous SCD (HbS/S).

#### 3.1.2. Enrolment of Screened Neonates with SCD into Comprehensive Care

**Figure 2:**
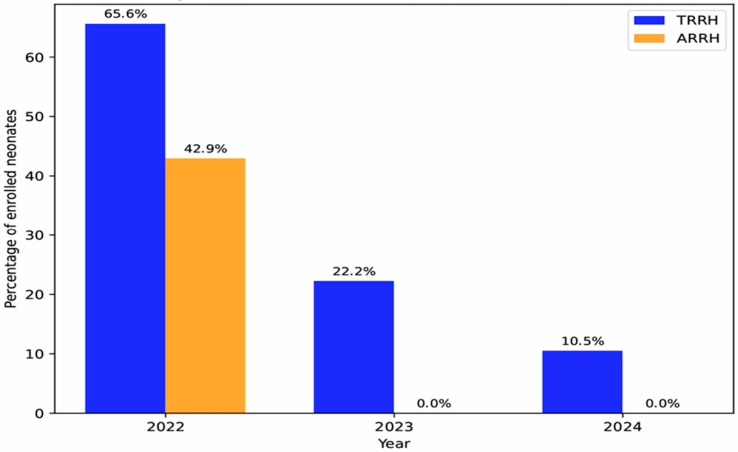
Proportion of neonates diagnosed with SCD and enrolled in comprehensive care, 2022–2024.

The proportion of neonates diagnosed with SCD who were subsequently enrolled in care declined across both facilities. At TRRH, enrolment declined from 65.6% in 2022 to 22.2% in 2023 and 10.5% in 2024. At ARRH, enrolment dropped from 42.9% in 2022 to 0% in 2023 and 2024 (Figure 2).

### 3.2. Qualitative Findings

A total of 17 nurses participated in the KIIs, where 10 came from TRRH and 7 from ARRH. Most participants were aged below 50, accounting for 70% at TRRH and 86% at ARRH. Female nurses predominated at both sites, representing 90% of participants at TRRH and 57% at ARRH. Participants’ educational backgrounds varied, including certificate holders (4 at TRRH, 3 at ARRH), diploma holders (5 at TRRH, 2 at ARRH), and degree holders (1 at TRRH, 2 at ARRH), reflecting a balance of practical experience and formal training. All participants were professional nurses, underscoring their central role in the implementation and delivery of the NBS program for SCD (Table 1).

#### 3.2.1. Facilitators of Implementation

Five main facilitators supported program implementation: pre-screening education, cross-departmental collaboration, universal screening policy, trained nurse performers, and parental engagement.

##### Pre-Screening Education

Health workers described pre-screening education as an essential part of the NBS process. They reported that mothers are given group counselling sessions upon arrival, where they are taught about SCD, its effects, and the importance of early diagnosis. The sessions also cover other newborn health topics such as breastfeeding and danger signs. These education sessions are intended to ensure that mothers understand the purpose of screening and how it can benefit their children.

*“Here with us, usually when mothers arrive, maybe one comes alone but what we do is we first conduct group counselling, we explain to them together so that they can understand what sickle cell disease is and what effects it has on children and even adults, and we also explain the importance of early testing, so that if the child is diagnosed early, steps can be taken. So, we teach them in the form of health education.”*

(KII 1, ARRH)

##### Cross-Departmental Collaboration

Strong interdepartmental collaboration was frequently cited as another critical facilitator. Coordination between departments enabled the reallocation of supplies such as testing kits when shortages occurred, ensuring uninterrupted service. Reproductive and Child Health staff also contributed significantly by guiding informed mothers through the process, demonstrating a unified effort in program execution. This cooperation reduced workflow delays and maintained service continuity during times of resource constraints. The synergy between departments served to enhance overall program effectiveness.

*“…other departments like our colleagues, after they deliver, they call us, saying come and take this baby, take the sample. They call us. Sometimes they find that the mother has a family history, for example, the mother has said there is sickle cell, so they help us. The mother asks for the baby to be tested for sickle cell.”*

(KII 1, TRRH)

##### Universal Screening Policy

The practice of testing every newborn without exception streamlined service delivery and minimized selection bias. Health workers emphasized the importance of universal coverage in ensuring equitable healthcare access and preventing missed diagnoses. Screening was initiated immediately after birth, often within the first 24 h, ensuring the timely identification of affected infants. This approach also facilitated routine integration into postnatal care workflows. The goal of same-day testing contributed to higher efficiency and better health outcomes.

*“…… we do not choose. Every mother who comes with a baby, we provide the education, and if she agrees, we test. We do not select a certain type of baby to be tested or not. Every mother who gives birth all babies should be tested.”*

(KII 2, TRRH)

##### Trained Nurse Performers

The presence of specifically trained nurses was essential in ensuring the quality and accuracy of the sample collection process. Most facilities had designated personnel trained through initial workshops and peer led inward mentoring. These nurses collaborated closely with ward in-charges to carry out screening activities effectively. The standardization of training helped maintain consistency across facilities. The continued emphasis on skill development contributed to a more robust and reliable screening process.

*“…… eight nurses were trained, and other nurses are under them, but mostly here in the neonatal ward, it’s those with the skills. However, there are like three nurses whom we trained internally, we trained them ourselves so that a child doesn’t miss the opportunity for screening.”*

(KII 1, ARRH)

##### Parental Engagement

The engagement of parents, particularly after thorough education sessions, contributed positively to the uptake of screening. Parents were generally receptive, frequently asked about timelines and follow up procedures, and demonstrated strong interest in understanding the screening’s implications. While occasional hesitations occurred, these were often addressed through repeated counselling and group persuasion techniques. The overall responsiveness of parents fostered a collaborative atmosphere. Sustained parental involvement also encouraged compliance with follow-up protocols.

*“Parents respond positively to this program after we educate them that it is important and it is free with no cost, this helps you to know your child from early on for clinic and examination, and to be given more education on how to care for the sick child. Very few decline but the response is very good, especially when they receive education.”*

(KII 3, TRRH)

#### 3.2.2. Challenges in Implementation

The challenges were identified based on participants’ experiences and perspectives shared during key informant interviews: inadequate training, delayed staff motivation, supply shortages, staff shortages, and parental hesitancy.

##### Inadequate Training

A significant barrier to effective implementation was the limited availability of refresher and seminar training sessions. Staff frequently expressed a desire for ongoing education to remain updated on screening protocols and best practices. The absence of continuous learning opportunities resulted in knowledge gaps and inconsistencies in service delivery. This limitation hindered the ability to sustain high-quality screening efforts. As a result, there was a call for regular, structured training programs.

*“The training is enough, but maybe if we had refreshers, it would help. I say this because, as I said, there are those who are lax… So if you came and repeated even a short training, if you gathered people and took them through it once again, it would create motivation.”*

(KII 1, ARRH)

##### Delayed Staff Motivation

Staff motivation was adversely affected by delays in incentive payments and unacknowledged efforts, even after resubmission of the data. Many health workers reported feeling demoralized when payments were delayed or withheld due to data entry errors, despite fulfilling their responsibilities. These issues created resentment and reduced enthusiasm for participating in the program. Prompt and transparent reward systems were seen as necessary for maintaining staff morale. Delayed compensation eroded trust and engagement.

*“Another challenge is the motivation for those collecting the samples they can go a long time without receiving anything, so some lose the morale to continue.”*

(KII 1, TRRH)

##### Supply Shortages

Irregular supply deliveries, stock-outs, and occasional equipment unavailability were among the most persistent challenges identified. Such disruptions led to idle periods where trained staff could not perform screenings, wasting both human and material resources. The resulting service interruptions weakened trust among caregivers and reduced the program’s credibility. Consistent supply chain management was flagged as a key area needing urgent attention. Gaps in supply coverage hindered timely diagnosis.

*“Getting supplies on time is a big challenge. You might finish your assigned 50 tests and then have to wait until others also finish before you get more kits.”*

(KII 5, TRRH)

##### Shortage of Staff

Facilities faced critical staff shortages, causing nurses to work outside duty hours and limiting screening capacity. The additional workload contributed to staff burnout and reduced commitment. High turnover rates further compounded the problem by necessitating repeated training cycles for new staff. Documentation burdens also increased, detracting from time spent with patients. These cumulative issues hampered the program’s efficiency.

*“ …. You may find that leaders are often busy with meetings. You find that in the ward there are only three nurses. It’s a challenge of staff shortage; you have a lot of work. So, at that time, even if you do it, you often do it in your extra time. After finishing your own duties, that’s when you do it.”*

(KII 7, ARRH)

##### Parental Hesitancy

Parental consent was sometimes delayed due to fears surrounding the heel-prick procedure or the need for husband approval. Misconceptions related to family history and cultural beliefs also played a role in hesitancy. In certain cases, mothers postponed giving consent until they had consulted family members, delaying the screening timeline. Ward transfers further complicated matters by requiring repeated education. Overcoming these barriers demanded intensive communication efforts.

*“They completely refused. They said, “No. If my child gets sick, I will take them to the hospital.” We told her, “But when the child starts getting sick, and you later suspect it might be sickle cell, you will have spent a lot of money. It’s better to know early and take steps before things get worse.” Among the three, two agreed to test, one refused completely, and we didn’t take the sample.”*

(KII 2, TRRH)

#### 3.2.3. Recommendations for Improvement

Healthcare workers provided recommendations for enhancing the implementation of the newborn screening program. These suggestions were based on their firsthand experience with the program and aimed at addressing existing gaps and improving service quality and sustainability: service integration, training expansion, incentive improvements, community outreach education, and supply chain strengthening.

##### Service Integration

There was a strong recommendation to integrate NBS into routine newborn services and postnatal check-ups. By embedding the service into existing workflows, health workers believed screening could become a standard part of postnatal care. Integration would also allow for more efficient use of time and resources while ensuring every eligible infant is reached. Additionally, suggestions were made to include screening in outpatient and inpatient services. Service integration was viewed as essential for scalability.

*“Maybe during postnatal check-up, when filling the form, I think it can help if we include this screening so the children can be screened. Because during the postnatal check-up, aah, you look at the mother but also you examine the baby. Right there we can also slip in matters of sickle cell, especially when giving health education, because the mother does not leave before receiving health education”*

(KII 3, TRRH)

##### Training Expansion

Expanding training efforts to include more staff was proposed as a means to increase program reach and sustainability. Participants emphasized the need for regular classroom training not only for screening personnel but also for surgical and support teams. The goal was to develop a workforce capable of maintaining program continuity despite turnover or absenteeism. This recommendation also included calls for peer-to-peer mentorship. A decentralized training approach was encouraged to build internal capacity.

*“Maybe more people should be available, be available and have this knowledge of testing these children, this sickle cell thing, yani each person should have this knowledge or if possible, you should prepare a strategy for people to receive classroom training, even if it’s just a one-day refresher, for a few hours, so that everyone has this knowledge.”*

(KII 3, TRRH)

##### Incentive Improvements

Improving the financial incentive structure was another frequently mentioned recommendation. Suggestions included implementing performance-based payments directly into staff accounts to avoid bureaucratic delays. It was believed that transparent and timely compensation would enhance staff commitment and morale. Higher incentives were seen as a motivating factor for better service delivery. Financial recognition was key to sustaining workforce engagement.

*“Improvements like we’ve said address the shortages, and provide motivation to those doing this work motivate them.”*

(KII 1, TRRH)

##### Community Outreach Education

Outreach efforts that targeted communities before hospital visits were encouraged to improve maternal readiness and streamline in-hospital screening. Participants proposed strategies akin to HIV outreach programs, including antenatal counselling and public health education. These efforts would aim to reduce hesitancy and promote informed decision-making. Early intervention was deemed critical in overcoming cultural and informational barriers. Decentralized education was viewed as a proactive solution.

*“Maybe a policy for the service to reach more widely. Maybe they should be visited at home or when they come to the hospital, they should be told about it about screening their children. That is, when children are born, they should be screened, but they should be informed, because some don’t know, and even when told, they don’t understand well.”*

(KII 6, ARRH)

##### Supply Chain Improvements

To mitigate recurring stock-outs, it was recommended that sickle cell screening kits be formally included in labour ward supply plans. Improving the predictability and reliability of supply deliveries was seen as essential to uninterrupted service. Stable stock availability would ensure efficient workflow and reinforce caregiver trust. Participants highlighted that planning at the policy level would yield long-term benefits. Strengthening the supply chain would reduce idle time and service gaps.

*“First, I would suggest that the ministry collaborate with those stakeholders. If they unite and join forces and we get that equipment supplied here at the hospital, it would help us a lot.”*

(KII 1, TRRH)

## 4. Discussion

This study provides important insights into the implementation of NBS for SCD in regional referral hospitals in Dar es Salaam, Tanzania. The implementation of NBS for SCD at Temeke and Amana Regional Referral Hospitals demonstrated early feasibility, with strong screening coverage supported by motivated staff, adequate resources, and interdepartmental collaboration. However, sustainability was undermined over time by systemic barriers, including supply shortages, inadequate training, delayed incentives, and weak referral and follow-up systems. Limited caregiver awareness and persistent community misconceptions further constrained enrolment into comprehensive care. To our knowledge, this is one of the first evaluations in the country to combine quantitative trends and qualitative perspectives in assessing the operational performance, facilitators, and challenges of NBS integration into routine maternal and child health services. Findings revealed strong uptake during the initial phase of implementation, followed by a decline in screening coverage and enrolment into comprehensive care, with health system and caregiver-related factors shaping program outcomes. These findings underscore both the promise and fragility of NBS programs in resource-limited settings, and calls for concerted efforts to update provider training curricula, strengthen community education, develop national guidelines and standard operating procedures, allocate budgets for NBS services and integrate NBS into routine maternal and child health services in order to improve the coverage, acceptability and success of NBS in Tanzania.

A key quantitative finding was the high proportion of neonates screened in the first year of program implementation. This mirrors early successes reported in Nigeria and Uganda, where donor support, motivated staff, and political commitment boosted initial coverage [14,15]. However, screening coverage declined significantly over time in both hospitals, consistent with experiences from Ghana and Kenya, where irregular supply chains, staff shortages, and limited government integration undermined long-term sustainability [16,17]. The interruptions in supply of test kits and irregular staffing reported in this study confirm that fragile health system structures threaten continuity of services. Strengthening procurement systems, embedding NBS supplies into essential medicines lists, and addressing human resource planning are critical steps for sustainability.

Equally concerning was the steep decline in enrolment of diagnosed newborns into comprehensive care. At Temeke, enrolment fell from over 65% in year one to just 10.5% in year three, while Amana recorded no new enrolments beyond the first year. Similar challenges have been documented across Sub-Saharan Africa, where weak referral pathways, poor caregiver awareness, and transportation barriers contribute to high attrition [18,19]. In Kenya, integration of SMS reminders and community health volunteer follow-up improved retention [4], while targeted caregiver education in Ghana enhanced linkage to care [20]. These findings suggest that Tanzania’s NBS program could benefit from digital reminder systems, strengthened referral pathways, and community-based linkage models.

Qualitative findings highlighted multiple facilitators of implementation. Pre-screening education and counselling sessions empowered mothers to make informed decisions and enhanced participation, similar to evidence from Uganda [21]. Interdepartmental collaboration between maternity, laboratory, and Reproductive and Child Health units promoted timely identification and follow-up, echoing lessons from Kenya [14]. Universal screening policies reduced missed diagnoses and promoted equity, while trained nurses ensured quality sample collection. Additionally, parental engagement and early timing of screening enhanced program acceptability. These enablers demonstrate that, even in resource-limited settings, strong institutional structures and staff dedication can drive effective implementation.

Despite these strengths, several barriers hindered program continuity. Providers reported inadequate refresher training, leaving knowledge gaps and reducing consistency in service delivery, an issue also observed in Nigeria and Ethiopia [22]. Low staff motivation due to delayed incentives contributed to demoralization, undermining screening efforts, while persistent supply shortages disrupted continuity and eroded caregiver trust, as similarly reported in Uganda and Zambia [23]. Staffing constraints and competing workload pressures reduced screening capacity, while parental hesitancy driven by fear, cultural beliefs, and spousal decision-making delayed consent, reflecting barriers noted across African NBS settings [24,25]. Addressing these challenges requires systemic solutions, including continuous professional development, transparent incentive structures, resilient supply chains, adequate staff allocation, and culturally tailored community engagement.

The strengths of this study include the use of a process evaluation design with mixed methods, which allowed triangulation of quantitative patterns with qualitative perspectives from frontline providers, enhancing validity and depth. The involvement of two high-volume urban hospitals increases applicability to similar settings in Tanzania, while the rigorous qualitative analysis ensured credibility and dependability of findings. However, limitations must be acknowledged. The reliance on secondary facility data may have introduced inaccuracies due to incomplete or inconsistent records. The focus on urban hospitals limits generalizability to rural or primary care settings, which may face different implementation challenges. In addition, the cross-sectional design restricts assessment of long-term outcomes, and some participants may have withheld candid views on sensitive management issues, potentially limiting depth of insights. Future studies should adopt prospective, longitudinal approaches, expand to rural facilities, and strengthen data systems for more accurate monitoring.

Overall, this study demonstrates that while NBS for SCD is feasible in Tanzania and initially achieves high uptake, its sustainability and impact are undermined by systemic and operational challenges. The findings highlight persistent gaps across the screening-to-care continuum, characterized by unstable screening coverage and progressively weaker linkage to comprehensive care following SCD diagnosis with more pronounced and persistently low screening coverage at Amana. Addressing supply chain resilience, staff training and motivation, caregiver education, and referral mechanisms will be essential to improve coverage and linkage to care. In order of priority, scaling up the program will require stronger government ownership, availability of diagnostics, integration of the services into national maternal and child health services, and investment in community engagement and digital innovations to ensure no child with SCD is missed. Furthermore, establishing robust monitoring and evaluation frameworks, supported by digital tools, will enhance data quality and implementation fidelity. Future research should examine caregiver perspectives, cultural determinants of participation, and the cost-effectiveness of NBS to guide national scale-up. With sustained government commitment and strategic investment, NBS for SCD can be institutionalized as a lifesaving intervention, ensuring timely diagnosis, early linkage to care, and improved health outcomes for affected children in Tanzania.

## Figures and Tables

**Table 1:** Sociodemographic characteristics of KII participants.

		TEMEKE RRH	AMANA RRH
	Total interviewed	10	7
Age	≤50 years	7	6
	>50 years	3	1
Sex	Male	1	2
	Female	9	5
Education level (Nurses)	Certificate	4	3
	Diploma	5	2
	Degree	1	2

## Data Availability

The data used and/or analysed during the current study are available from the corresponding author on reasonable request.
